# Correlation of Eustachian tube function with the results of type 1 tympanoplasty: a prospective study

**DOI:** 10.1007/s00405-022-07611-4

**Published:** 2022-08-26

**Authors:** Waleed Moneir, Noha Ahmed El-Kholy, Ahmed Ismail Ali, Mohamed Moustafa Abdeltawwab, Asser Abdel Raouf El-Sharkawy

**Affiliations:** 1 Department of Otolaryngology-Head and Neck Surgery, Faculty of Medicine, Mansoura University, Mansoura, Egypt; 2 Department of Audiology, Faculty of Medicine, Mansoura University, Mansoura, Egypt

**Keywords:** Tympanoplasty, Eustachian tube, Graft take rate, Saccharine test, Pressure equalization test

## Abstract

**Objectives:**

This study aims to evaluate Eustachian tube (ET) function tests and their impact on outcomes of tympanoplasty in patients with inactive chronic suppurative otitis media.

**Materials and methods:**

A prospective study was conducted involving patients diagnosed with chronic suppurative otitis media (CSOM) and having a central dry perforation. Assessment of the ET function was done for all included cases by three tests; pressure swallow equalization test, saccharine test and methylene blue test. The primary outcome is the graft success rate defined as intact graft without any residual perforation at 6 months postoperatively. Secondary outcomes include hearing assessment and possible associated complications.

**Results:**

64 patients were included in the study with an average age of 36.59 ± 11.96 years. All patients underwent assessment of the ET function by saccharine test, methylene blue test and pressure equalization test (PET) followed by microscopic post-auricular tympanoplasty. Successful tympanoplasty is achieved in 93.75% of cases with residual perforation in four patients. Mean air–bone gap is significantly improved from 23.73 ± 2.80 preoperatively to 10.93 ± 5.46 postoperatively. Results of Methylene blue test has no statistical impact on graft take rate (*p* value = 0.379), while saccharine test and pressure equalization test results have statistically significant correlation with graft success (*p* value ≤ 0.001).

**Conclusions:**

Saccharine and Pressure equalization tests have a good positive correlation with the graft healing in tympanoplasty, while methylene blue test was found to have no correlation with the success rate.

## Introduction

The Eustachian tube (ET) is a connecting channel between the tympanic cavity and the nasopharynx and has three main functions; maintaining the middle ear pressure, drainage of the middle ear secretions by mucociliary clearance and protection of the middle ear from retrograde reflux of the nasopharyngeal sounds and pathogens [1].

ET dysfunction plays an important role in the pathogenesis of otitis media and subsequently in the success rate of tympanoplasty. Thus, assessment of the ET function has been the center of focus of many studies [2]*.* There is conflict between authors about role of ET in success rate of tympanoplasty, some concluded that ET dysfunction has no effect on the outcome of tympanoplasty, while others insisted on its important role [3]. This lack of consensus may be due to absence of a single gold standard test that can measure the ET function accurately and its relation to tympanoplasty [4]. Thus, combination of the ET function tests may be helpful in minimizing this conflict and predicting possible surgery failure. The present study aims to evaluate ET function and its impact on surgical outcomes in tympanoplasty patients.

## Patients and methods

### Study design

A prospective study was conducted in the Otorhinolaryngology Department during a 1-year period, between November 2019 and October 2020, involving patients diagnosed with chronic suppurative otitis media (CSOM). Included cases must have a small or medium-sized perforation with dry ear for at least 3 months before surgery and their age should range from 18 to 60 years. Patients with active ear discharge and those with incomplete data or lost follow-up were excluded from the study. Also, the following conditions were not included in this study; patients having associated ossicular abnormalities, recurrent perforations after mastoidectomy or tympanoplasty and cases with total and subtotal perforations. Ethical approval was obtained from our institutional review board (MS.19.11.886) and written informed consent was taken from included patients.

Basic demographic data were collected for the studied subjects. A 4 mm 0° (Karl Storz, Germany) endoscope was used to determine site and size of the TM perforation, examine ossicles, through the perforation, middle ear mucosa, nasopharynx and ET opening. Tuning fork tests (Rinne and Weber tests) were applied to the cases.

Preoperative pure tone audiometry, tympanometry and speech audiometry results were reported. The threshold of air conduction (AC) and bone conduction (BC) were obtained at 0.5, 1, 2, 4 kHz frequencies by supra aural headphone for AC and bone vibrator for BC. The mean air bone gab (ABG) was calculated as the difference between both.

Assessment of the ET function was done by three tests; pressure swallow equalization test, saccharine test and methylene blue test.

**Fig. 1:**
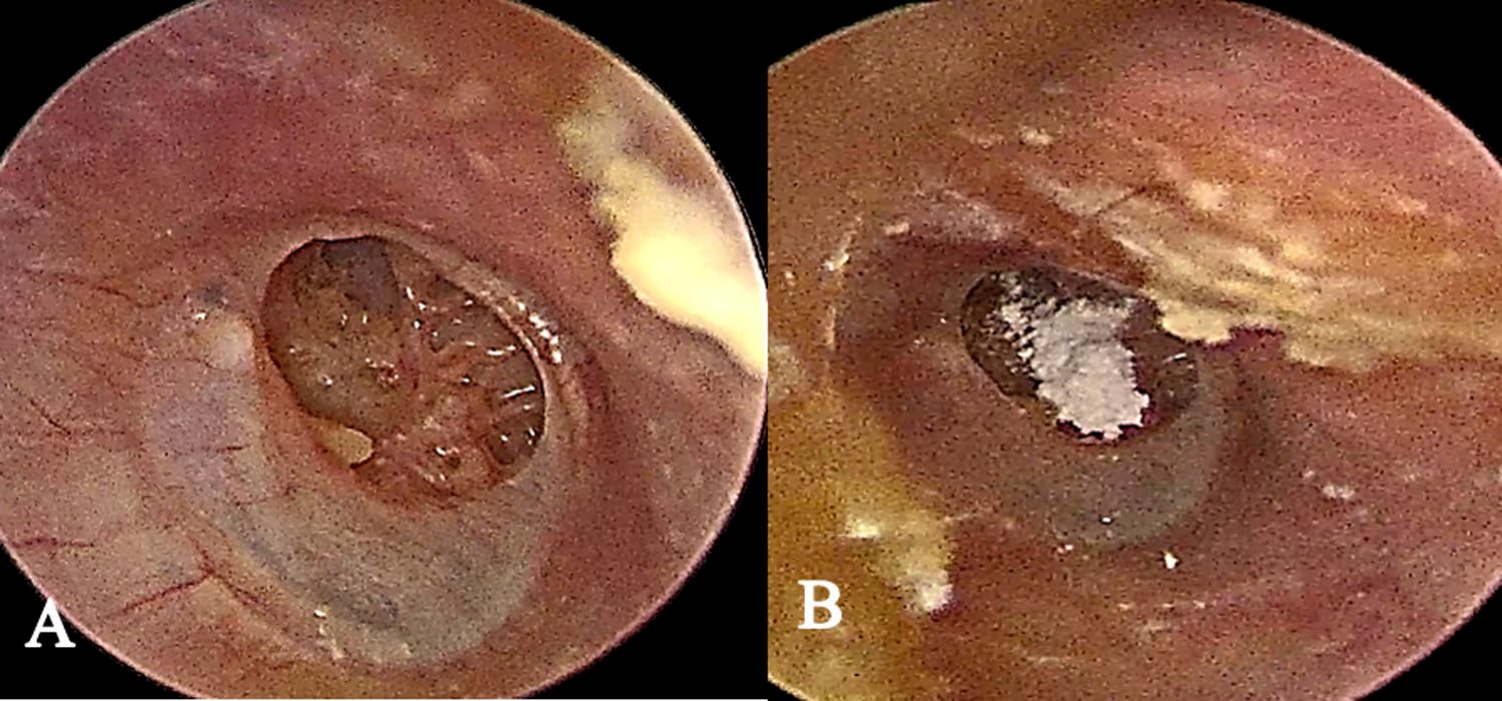
Rt medium-sized anterior tympanic membrane perforation **A** before and **B** after saccharin placement

**Fig. 2:**
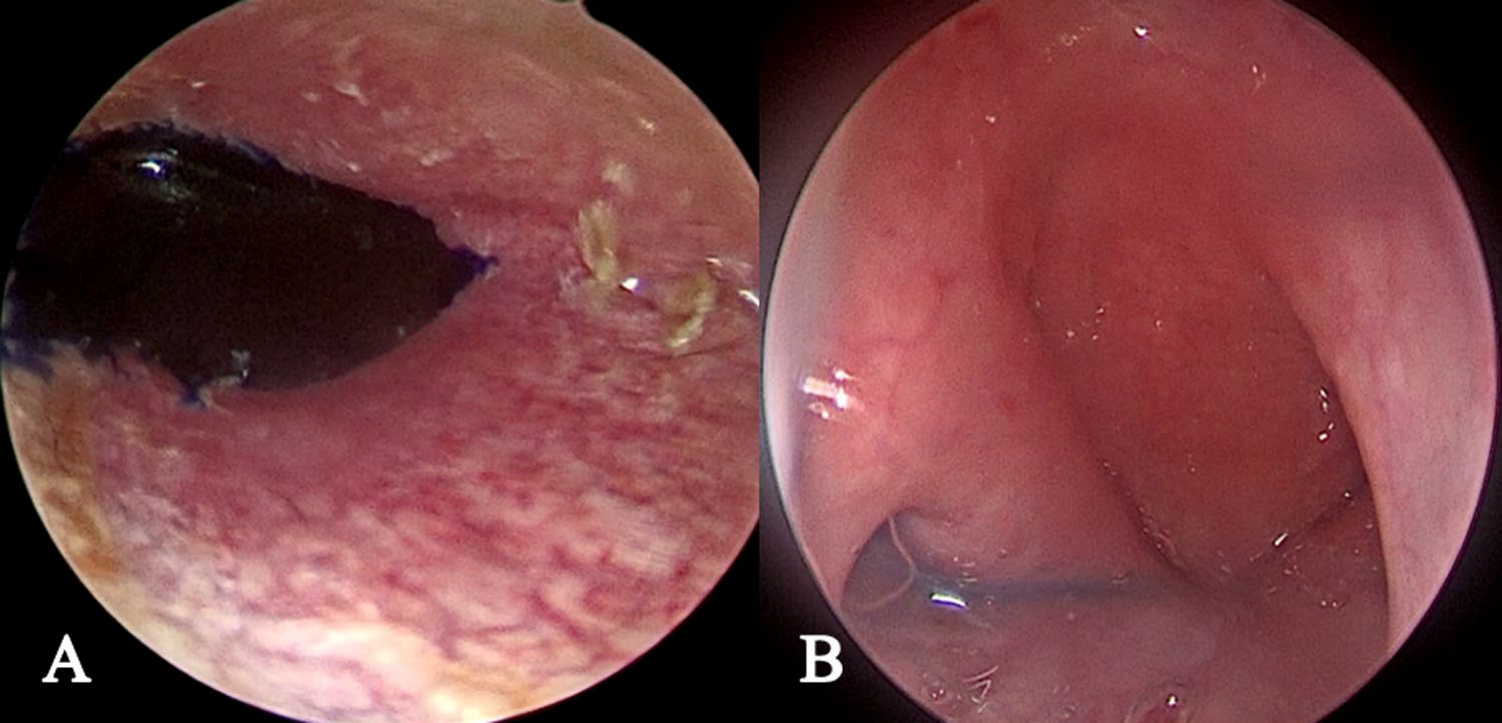
Methylene blue test: **A** shows instillation of the methylene blue into the middle ear, **B** shows detection of methylene blue at the nasopharyngeal opening of the Eustachian tube

- Pressure swallow equalization test:
  This test is done using Interacoustic Titan Tympanometry. After selection of the appropriate probe tip, a negative middle ear pressure was created down to − 200 mmH_2_O. Then, the patient was instructed to swallow three to five times until no pressure changes to calculate the residual negative pressure (RP) and physical volume [5]. Accordingly, patients were divided into three groups; Good ET function, Partial ET dysfunction and Gross ET dysfunction, where good means physical volume > 2.5 ml (RP 0–50), partial dysfunction means physical volume 2–2.5 ml (RP 50–150) and gross dysfunction means physical volume 1.5–2 ml (RP 150–200).
- Saccharine test:
  This test is used to assess mucociliary clearance function of the ET. It is performed on the same day of the surgery using saccharine powder. The powder was placed in the tympanic cavity through the TM perforation. The amount used is that filling ear curette twice (Fig. 1). Then, the patient remains in the sitting position and instructed to indicate the time at which sweet taste is noticed. The time required by the patient to have a sweet taste is measured, saccharine perception time (SPT). If the patient does not report a saccharine taste after 45 min, saccharine is placed on the tongue. If this test was also negative, the result was noted as no response and the patient is excluded from the study. According to SPT, patients are divided into three groups; Good ET function: SPT less than 20 min, Partial ET dysfunction: SPT was 20–45 min, Gross ET dysfunction: SPT > 45 min [6].
- Methylene blue test:
  In the same setting, after clearance of the residual saccharine powder by suction, two to three drops of sterile methylene blue dye 1%, diluted with saline, were instilled in the tympanic cavity through the perforation in the sitting position. Using 4 mm diameter, 0° nasal endoscope, the nasopharynx is examined for the dye at the ET opening after 10 min (Fig. 2). If no dye is encountered, examination is repeated every 10 min up to half an hour. The patients are divided into three groups according to methylene transport time [6]; Good ET function: less than 10 min, Partial ET dysfunction: from 10 to 20 min and Gross ET dysfunction: more than 20 min.

### Surgical technique

All patients underwent tympanoplasty under general anesthesia by two highly experienced surgeons through microscopic post-auricular approach with over-underlay technique using temporalis fascia graft.

**Fig. 3:**
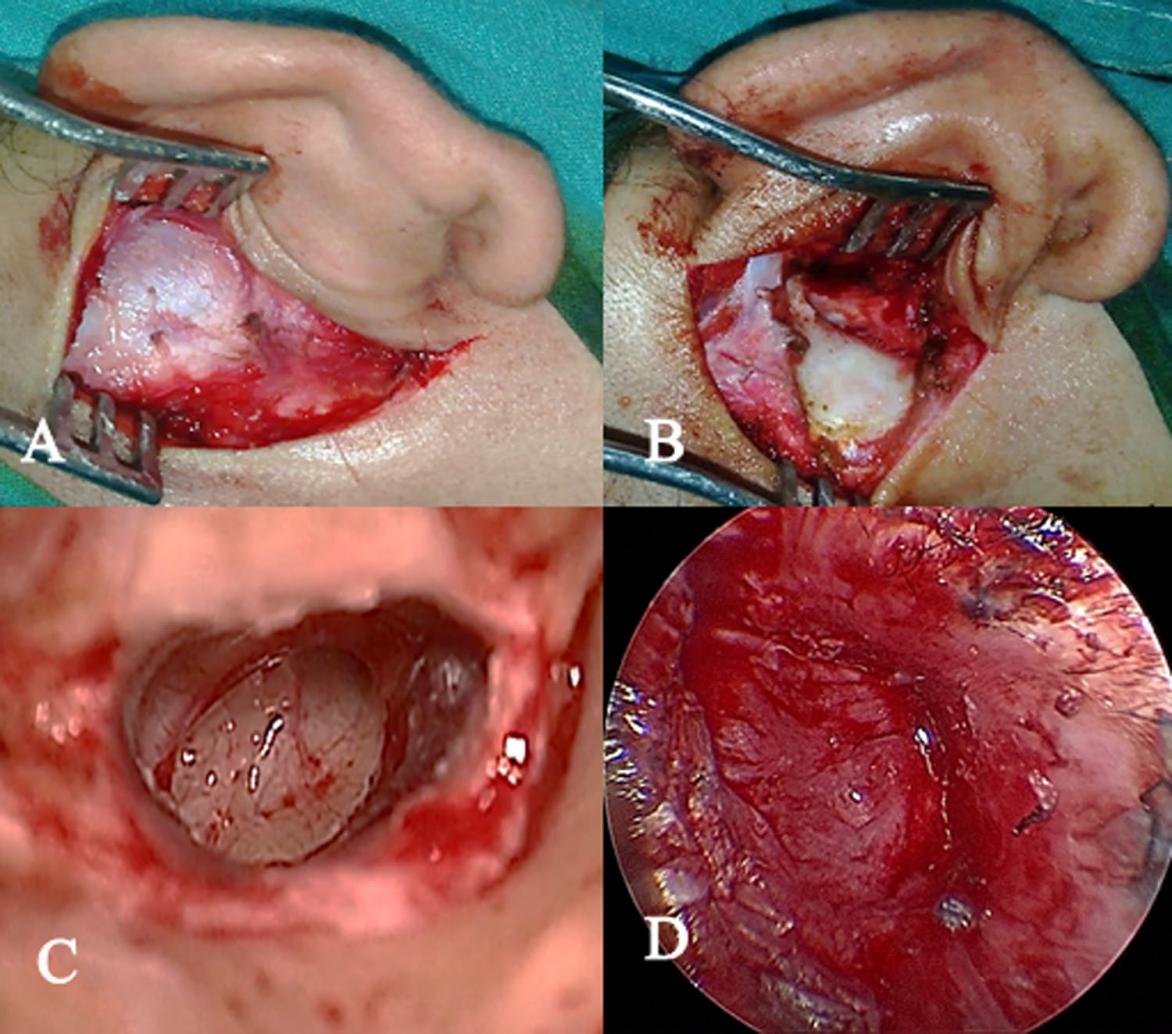
Steps of tympanoplasty; **A** exposure of the temporalis fascia for graft harvest, **B** elevation of mucoperiosteal flap, **C** right tympanic membrane after refreshment, **D** placement of the temporalis fascia graft

The surgery starts with the patient in supine position with the head tilted to the contralateral side. After surgical sterilization and draping, microscopic clearance of any methylene blue residual is done. Local infiltration with diluted adrenaline (1/100,000) is followed by a post-auricular incision made 0.5 cm from the post-auricular sulcus. The superficial layer of the temporalis fascia is harvested for grafting preserving the deep layer in case a revision surgery is required. Then, fascia is dried and pressed. An anteriorly based mucoperiosteal flap is elevated and then an incision is made in the posterior meatal skin (vascular strip) at the level of spine of Henle. Through this incision, refreshment of the margins of the TM perforation is done by curved needle. Elevation of tympanomeatal flap is done from 12 o’clock to 6 o’clock together with the posterior fibrous annulus with preservation of chorda tympani. Then, the tympanic cavity is examined, the ossicular chain mobility is tested and the ventilation pathways are evaluated for its patency. The middle ear is packed with Gelfoam to support the graft medially. The dried temporalis fascia graft is placed over the handle of malleus and under the annulus and tympanic membrane remnant (over-underlay technique). Once the graft is in good position, the tympanomeatal flap is returned to its original position. Then, the external auditory canal is packed with Gelfoam, the mucoperiosteal flap is closed with absorbable sutures and skin incision is closed in layers (Fig. 3).

### Postoperative care

The patients were discharged on the same day of the surgery, systemic antibiotics, analgesics and local otrivin nasal drops were prescribed for 1 week after the surgery. List of instructions was given to every patient in the form of rest and avoiding straining, sneezing and nose blowing.

### Follow-up

After 1 week, the dressing and sutures were removed and antibiotic–steroid ear drops are started for 1 week. Patients are advised to keep the operated ear dry and away from water. Determination of graft take was done by endoscopy 3 months postoperatively. This is repeated at 6 months postoperatively together with PTA and tympanometry.

### Outcome measures

The primary outcome is the graft take rate and graft success is defined as intact graft without any residual perforation determined by 0° endoscopic examination 6 months postoperatively. Secondary outcomes include hearing assessment done within 1 month before the surgery and at 6 months after the surgery. Postoperative complications are evaluated and include; infection, postoperative wound dehiscence, anterior blunting, graft lateralization, reperforation, cholesteatoma formation, tinnitus, hearing deterioration, canal stenosis, loss of taste and facial nerve affection.

### Statistical analysis

Data were analyzed using the Statistical Package of Social Science (SPSS) program for Windows (Standard version 21). The normality of data was first tested with one-sample Kolmogorov–Smirnov test. Qualitative data were described using number and percent. Continuous variables were presented as mean ± SD (standard deviation) for normally distributed data. Monte carlo test is used to compare qualitative variables when expected count less than 5. Paired *t* test is used to compare quantitative data preoperatively and postoperatively. ANOVA test compares more than two means (parametric) and Pearson correlation is used to correlate two continuous variables (parametric). For all above mentioned statistical tests done, results are considered significant when *p* ≤ 0.05.

## Results

This study includes 64 patients with inactive mucosal type CSOM (20 males, 44 females) with 64 operated ears (36 right, 28 left) who met the inclusion criteria mentioned before and were included in this study. Fifteen patients (23.5%) had a contralateral ear disease (atelectasis, secretory otitis media, adhesive otitis media, tympanosclerosis or inactive mucosal CSOM). The mean age was 36.59 ± 11.96 years ranged from 18 to 58 years with no significant difference in relation to the surgical outcome (*p* value = 0.479).

All patients underwent assessment of the ET function by saccharine test, methylene blue test and pressure equalization test (PET) followed by microscopic post-auricular tympanoplasty. Demographic and basic data are presented in Table 1. Results of ET function tests are demonstrated in Table 2. Successful tympanoplasty was achieved in 93.75% of cases with residual perforation in four patients. Mean ABG is significantly improved from 23.73 ± 2.80 preoperatively to 10.93 ± 5.46 postoperatively. Postoperative infection is encountered in two cases and loss of taste is reported in one case.

**Table 1:** Basic demographic and clinical data of the studied group

Parameter	The study group (*n* = 64)
Age (years) mean ± SD (min–max)	36.59 ± 11.96 (18–58)
Sex	
Male	20 (31.2%)
Female	44 (68.8%)
Site	
Anterior	24 (35.9%)
Posterior	17 (26.6%)
Central^3^	23 (35.9%)
Size	
Small	18 (28.1%)
Medium	19 (29.7%)
Large	27 (42.2%)
Side	
RT	36 (56.25%)
LT	28 (43.75%)
Contralateral ear status	
Normal	49 (76.56%)
Diseased	15 (23.44%)

*Anterior* TM perforation anterior to handle of malleus, *Posterior* TM perforation posterior to handle of malleus, *Central* TM perforation involved both sides of handle of malleus equally, *Small* limited to one quadrant, *Medium* involved two quadrant, *Large* involved three quadrants, Diseased contralateral ear; including atelectasis, adhesive otitis media, secretory otitis media, tympanosclerosis and CSOM

**Table 2:** Results of Eustachian tube function tests, graft take rate, hearing assessment and surgical complications on included patients

Parameters		The study group (*n* = 64)
Eustachian tube Function tests	PET	
	Normal	44 (68.8%)
	Partial dysfunction	10 (15.6%)
	Gross dysfunction	10 (15.6%)
	Saccharine perception time (SPT) (min)	
	(Mean ± SD)	18.80 ± 5.83
	Normal function	16.08 ± 1.86
	Partial dysfunction	28.85 ± 4.71
	Saccharine test	
	Normal	48 (75.0%)
	Partial dysfunction	13 (20.3%)
	Gross dysfunction	3 (4.7%)
	Methylene blue test	
	Normal	55 (85.9%)
	Partial dysfunction	9 (14.1%)
	Gross dysfunction	0 (0%)
Graft uptake	Healed graft (success)	60 (93.75%)
	Intact	53 (82.8%)
	Retracted	7 (10.9%)
	Residual perforation (failure)	4 (6.25%)
Hearing assessment	Mean ABG	23.73 ± 2.80
	Preoperatively	
	Postoperatively	10.93 ± 5.46
	*p* value	≤ 0.001*
Complications	No complications	61 (95.3%)
	Infection	2 (3.1%)
	Loss of taste	1 (1.6%)

*ABG* Air–bone gap, *PET* pressure equalization test, *SPT* saccharine perception time. For PET: good means physical volume > 2.5 ml (RP 0–50), partial dysfunction means physical volume 2–2.5 ml (RP 50–150), gross dysfunction means physical volume 1.5–2 ml (RP 150–200). For Saccharine test: good means SPT < 20 min, Partial dysfunction means SPT 20–45 min, Gross dysfunction: SPT > 45 min. For methylene blue test: good ET function: methylene transport time < 10 min, Partial ET dysfunction: from 10 to 20 min and Gross ET dysfunction > 20 min

Results of ET function by Methylene blue test has no statistical impact on graft take rate (*p* value = 0.379), while saccharine test and pressure equalization test results have statistically significant correlation with graft success (Table 3) (*p* value ≤ 0.001). Table 4 shows that patients with anterior perforations has the shortest SPT followed by patients with central perforation and the Longest SPT is detected in patients with posterior perforations with statistically significant difference. There is no statistically significant difference between different sites of perforation as regard methylene blue test and PET (*p* value = 0.908 and 0.175, respectively). None of the listed complications were reported postoperatively for the follow-up period.

**Table 3:** Correlation between Eustachian tube function tests and graft take rate

Test	Graft take rate			*p* value
	Intact	Retraction	Perforation	
Methylene blue test				
Normal (*n* = 55)	47 (85.45%)	5 (9.09%)	3 (5.46%)	0.379
Partial dysfunction (*n* = 9)	6 (66.67%)	2 (22.22%)	1 (11.11%)	
Gross dysfunction (*n* = 0)	0 (0%)	0 (0%)	0 (0%)	
Saccharine test				
Normal (*n* = 48)	44 (91.67%)	4 (8.33%)	0 (0%)	≤ 0.001*
Partial dysfunction (*n* = 13)	9 (69.23%)	3 (23.08%)	1 (7.69%)	
Gross dysfunction (*n* = 3)	0 (0%)	0 (0%)	3 (100%)	
PET				
Normal (*n* = 44)	43 (97.73%)	0 (0%)	1 (2.27%)	≤ 0.001*
Partial dysfunction (*n* = 10)	8 (80%)	1 (10%)	1 (10%)	
Gross dysfunction (*n* = 10)	2 (20%)	6 (60%)	2 (20%)	

*PET* pressure equalization test, *SPT* saccharine perception time. For PET: good means physical volume > 2.5 ml (RP 0–50), partial dysfunction means physical volume 2–2.5 ml (RP 50–150), gross dysfunction means physical volume 1.5—2 ml (RP 150–200). For Saccharine test: good means SPT < 20 min, Partial dysfunction means SPT 20–45 min, Gross dysfunction: SPT > 45 min. For methylene blue test: good ET function: methylene transport time < 10 min, Partial ET dysfunction: from 10 to 20 min and Gross ET dysfunction > 20 min

**Table 4:** Correlation between site of TM perforation and ET function tests

Eustachian tube Function tests	Anterior (*n* = 24)	Central (*n* = 23)	Posterior (*n* = 17)	*p* value
Methylene blue test				0.908
Normal	21 (87.5%)	20 (87.0%)	14 (82.4%)	
Partial dysfunction	3 (12.5%)	3 (13.0%)	3 (17.6%)	
Gross dysfunction	0%	0%	0%	
SPT (min)				0.005*
Mean ± SD	15.82 ± 1.37	20.13 ± 7.09	21.25 ± 6.31	
Saccharine test				0.013*
Normal	23 (95.8%)	16 (69.6%)	9 (52.9%)	
Partial dysfunction	0 (0%)	6 (26.1%)	7 (41.2%)	
Gross dysfunction	1 (4.2%)	1 (4.3%)	1 (5.9%)	
PET				0.175
Normal	19 (79.2%)	14 (60.9%)	11 (64.7%)	
Partial dysfunction	1 (4.2%)	4 (17.4%)	5 (29.4%)	
Gross dysfunction	4 (16.7%)	5 (21.7%)	1 (5.9%)	

*ABG* Air–bone gap, *PET* pressure equalization test, *SPT* saccharine perception time. For PET: good means physical volume > 2.5 ml (RP 0–50), partial dysfunction means physical volume 2–2.5 ml (RP 50–150), gross dysfunction means physical volume 1.5–2 ml (RP 150–200). For Saccharine test: good means SPT < 20 min, Partial dysfunction means SPT 20–45 min, Gross dysfunction: SPT > 45 min. For methylene blue test: good ET function: methylene transport time < 10 min, Partial ET dysfunction: from 10 to 20 min and Gross ET dysfunction > 20 min

## Discussion

The normal functioning ET is an integral part of the good aeration of the middle ear and good tubotympanic mucociliary clearance. It has been the center of focus as a prognostic factor for any middle ear surgery due to its important role in pathogenesis of otitis media [7]. However, there is no definitive gold standard test for assessment of ET function and there is lack of consensus regarding the significance of these tests before tympanoplasty [4].

There are many studies that assessed the pressure regulatory function of ET by tympanometry, Valsalva and Toynbee maneuvers or by a special device, such as sonotubometry [8], pressure equalization test [5, 9], tubomanometry [10], forced response test [11, 12] and pressure chamber [13]. Few studies assessed the mucociliary function of the ET using methylene blue [6, 14], saccharine [15, 16], fluorescein [17] or radioisotope [18].

Although tympanoplasty enjoys a high success rate up to 96%, residual perforation in the remaining cases or postoperative retractions and adhesions can be related to affected middle ear ventilation or ET dysfunction [19, 20]. In this study, the effect of ET dysfunction on graft take rate in tympanoplasty is investigated by pressure equalization test, methylene blue test and saccharine test.

In this study, all cases underwent tympanoplasty via microscopic postauricular approach which provided a binocular vision with an excellent magnification and has the advantage of two-hand surgery which help in better control in case of bleeding and providing better graft manipulation and secure fitting under the tympanic membrane remnant.

The over-underlay technique was used in the present study. It provides the advantages of the two most common tympanoplasty techniques, overlay and underlay techniques, with minimal disadvantages. The over-underlay technique is a simple technique, suitable for perforations of all sizes and sites with low risk of lateralization as the graft is put medially to the annulus and TM remnant and no reduction of the middle ear space as the graft put lateral to the handle of the malleus [21, 22]. All cases in the study were done using temporalis fascia only as a graft material as it is the most commonly used material for TM reconstruction with good postoperative outcomes [23–26].

In this study, the graft uptake rate was 93.75% (60 patients) with residual perforation in 6.25% (4 patients). Seven patients (10.9%) with successful graft uptake had tympanic membrane retraction or effusion. These results are comparable with the mean closure rate in tympanoplasty published before (86.6%) [25]. Also, Hsu and Kouhi, reported graft uptake rates with temporalis fascia of 92% and 91.6%, respectively[27, 28].

In saccharine test, the powder form is preferred as saccharine solution can gravitate through the ET to the nasopharynx [29] and saccharine tablet may be less dispersible [6, 15]. Regard the test results in the current study, normal ET function was found in 48 patients (75%), partial dysfunction in 13 patients (20.3%) and gross dysfunction in 3 patients (4.7%). the overall mean SPT was 18.80 ± 5.83 min with mean SPT in the normal group 16.08 ± 1.86 min and in the partial group 28.85 ± 4.71 min. These results are in accordance with other studies. The mean SPT reported by Prasad et al. [6] was 17.583 min, while Ikehata et al. [15] found that the mean SPT was 14 min and 30 min in normal and partial dysfunction groups, respectively. Successful grafting is obtained in 91.67% of patients with normal ET function and 69.23% of patients with partial dysfunction, while none of those with gross dysfunction had postoperative normal TM. Graft failure is reported in the three patients diagnosed with gross ET dysfunction and in one patient with partial dysfunction (*p* value ≤ 0.001).

Elbrond and Larsen, 1976, assessed the ET using saccharine crystals and found that mean SPT was 32 min longer than the mean SPT in the current study. This disparity in timing can be explained that they used saccharin crystals which were less soluble than saccharin powder[30].

As regard the methylene blue test, normal ET function was found in 85.94% of patients (55 patients), partial ET function in 14.06% (9 patients). None of patients had gross ET dysfunction indicating no included patients had anatomically blocked ET. Postoperative normal tympanic membrane was achieved in 85.45% and 66.67% of patients having normal ET function and partial ET dysfunction, by methylene blue test, respectively. 22.22% of patients with partial ET dysfunction and 9.09% of patients with normal ET function had healed graft, but with retraction. There is no significant difference between the normal and abnormal groups regarding graft take rate (*p* value = 0.379) indicating no correlation between methylene blue test and graft success rate. This is explained by the authors by the fact that methylene blue solution may gravitate through the ET and not being actively transported by mucociliary clearance.

As regard the pressure equalization test (PET), the normal ET function was found in 44 of 64 (68.8%) ears, partial ET dysfunction in 10 of 64 (15.6%) of ears and gross dysfunction in 10 of 64 (15.6%) of ears. In relation to the graft uptake rate, intact tympanic membrane without retraction was obtained in 97.27% of ears (43 ears) with normal ET function and in 80% of ears (8 ears) with partial ET dysfunction and in only two cases with gross ET dysfunction. However, associated retraction is encountered in six cases with gross dysfunction and only one case with partial dysfunction. These results shows statistical significant correlation between ET function assessment by pressure equalization test and the resulting graft take rate. These results are comparable to previous literature;

In 1993, Gimenez and Marco-Algarra [29] assessed the ET function using sodium saccharin solution and found a higher percentage of normal transport in successful group than in the failure group. Also, they observed that ears with posterior perorations had longer transport time than anterior perforations which is similar to what found in the present study.

Prasad et al., assessed the ET by methylene blue and saccharin test [6] and reported a success rate of 93.75% in patients with normal ET function and a rate of 68.42% in patients with partial dysfunction. Those with gross dysfunction had a 100% failure rate. Similar to the current study, they concluded that saccharin and methylene blue tests have a good predictive value for success rate of tympanoplasty. Also, Das et al. [16], had concomitant results.

In this study, the PET also had a direct relation with the surgical outcome. The results are comparable with the results of Mackinnon et al. [31] who used the same test and found that the success rate of tympanoplasty was 80% in patients with good or partial ET dysfunction and 29% in patient with gross dysfunction.

Dave and Ruparel, 2019, used the negative pressure equalization test to assess the ET function. They found that the success rate was 87% in patients with no or mild ET dysfunction, 80% in patients with mild dysfunction and 70% in patients with persistent moderate or severe ET dysfunction.

In this study, PET was also a good predictor for the post-operative middle ear aeration and the hearing improvement. About 60% of patients with gross dysfunction by PET and 10% of the patients with partial dysfunction had healed graft with retraction. Choi et al. [9] evaluated the ET function using modified pressure equalization test and found that residual postoperative TM perforations, type B tympanograms and the worst hearing outcomes were observed in ears showing no pressure changes of the applied pressure after swallowing.

As regard the hearing outcomes in this study, the mean preoperative ABG was significantly improved from 23.73 ± 2.81 dB to become 10.3 ± 5.46 dB postoperatively. Patients with intact tympanic membrane, without retractions, had a postoperative ABG of 9.07 ± 3.34 dB in contrast to those with retracted TM and those with persistent perforation who had a postoperative mean ABG of 20.57 ± 1.56 and 22.0 ± 2.39 dB, respectively. These results were similar to Ikehata et al. [15] who reported worse hearing outcomes in patients with gross and partial ET dysfunction by saccharin test.

High proportion of the patients with contralateral ear disease (atelectasis, adhesive otitis media, tympanosclerosis and secretory otitis media) in the current study showed ET dysfunction. Both Eustachian tubes are symmetrical so the contralateral ear status can be used as indicator for ET function and predictor of tympanoplasty outcome. There were similar findings in previous studies [20, 32, 33]. Conversely, Singh and others, 2005, found no association between the status of the contralateral ear and surgical outcomes.

Saccharin and methylene blue tests showed no side effects, none of cases developed worsening of the preoperative hearing only one case had mild irritation with methylene blue. All previous studies used the saccharin and methylene blue reported no side effects [5].

Limitations of the study are the short follow-up period and relatively small sample size.

## Conclusions

There is a correlation between the ET function and the results of tympanoplasty. Saccharin test and Pressure equalization test showed a good positive correlation with the graft healing and the hearing outcomes in contrary to methylene blue test which has no correlation with the surgical outcome.
